# The Genetic Basis, Lung Involvement, and Therapeutic Options in Niemann–Pick Disease: A Comprehensive Review

**DOI:** 10.3390/biom14020211

**Published:** 2024-02-11

**Authors:** Claudio Tirelli, Ornella Rondinone, Marta Italia, Sabrina Mira, Luca Alessandro Belmonte, Mauro De Grassi, Gabriele Guido, Sara Maggioni, Michele Mondoni, Monica Rosa Miozzo, Stefano Centanni

**Affiliations:** 1 Respiratory Unit, ASST Santi Paolo e Carlo, Department of Health Sciences, University of Milan, 20142 Milan, Italy; marta.italia@unimi.it (M.I.); sabrina.mira@unimi.it (S.M.); luca.belmonte@unimi.it (L.A.B.); mauro.degrassi@unimi.it (M.D.G.); gabriele.guido@unimi.it (G.G.); sara.maggioni@unimi.it (S.M.); michele.mondoni@unimi.it (M.M.); stefano.centanni@unimi.it (S.C.); 2 Medical Genetics Unit, ASST Santi Paolo e Carlo, Department of Health Sciences, University of Milan, 20142 Milan, Italy; ornella.rondinone@unimi.it (O.R.); monica.miozzo@unimi.it (M.R.M.)

**Keywords:** Niemann–Pick Disease, acid sphingomyelinase deficiency, SMPD1, NPC1, NPC2, Olipudase α, miglustat, lung transplant

## Abstract

Niemann–Pick Disease (NPD) is a rare autosomal recessive disease belonging to lysosomal storage disorders. Three types of NPD have been described: NPD type A, B, and C. NPD type A and B are caused by mutations in the gene *SMPD1* coding for sphingomyelin phosphodiesterase 1, with a consequent lack of acid sphingomyelinase activity. These diseases have been thus classified as acid sphingomyelinase deficiencies (ASMDs). NPD type C is a neurologic disorder due to mutations in the genes *NPC1* or *NPC2*, causing a defect of cholesterol trafficking and esterification. Although all three types of NPD can manifest with pulmonary involvement, lung disease occurs more frequently in NPD type B, typically with interstitial lung disease, recurrent pulmonary infections, and respiratory failure. In this sense, bronchoscopy with broncho-alveolar lavage or biopsy together with high-resolution computed tomography are fundamental diagnostic tools. Although several efforts have been made to find an effective therapy for NPD, to date, only limited therapeutic options are available. Enzyme replacement therapy with Olipudase α is the first and only approved disease-modifying therapy for patients with ASMD. A lung transplant and hematopoietic stem cell transplantation are also described for ASMD in the literature. The only approved disease-modifying therapy in NPD type C is miglustat, a substrate-reduction treatment. The aim of this review was to delineate a state of the art on the genetic basis and lung involvement in NPD, focusing on clinical manifestations, radiologic and histopathologic characteristics of the disease, and available therapeutic options, with a gaze on future therapeutic strategies.

## 1. Introduction

**Figure 1:**
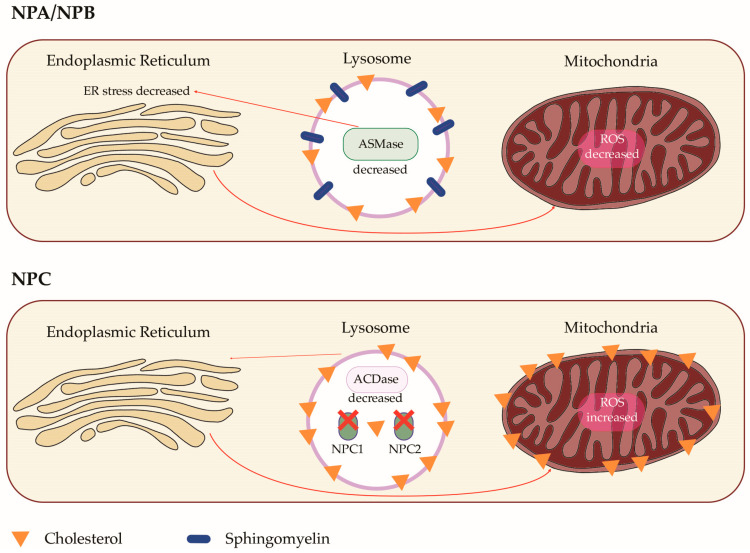
Molecular pathogenetic mechanisms in ASMD (NPD type A and B) and NPD type C. ASMDs (NPD type A and B) are due to acid sphingomyelinase (ASMase) deficiency. This leads to accumulation of sphingomyelin in lysosomes. As a consequence, a secondary sequestration of cholesterol in lysosomes occurs. ASMase acts as a trigger for endoplasmic reticulum stress. The consequent reduced endoplasmic reticulum stress signaling causes a decrease in reactive oxygen species (ROS) in mitochondria. NPD type C is characterized by defects of NPC1 and NPC2. This leads to accumulation of cholesterol in lysosomes, which is sustained by decreased activity of acid ceramidase (ACDase). As a consequence, via a poorly understood mechanism involving endoplasmic reticulum signals, mitochondrial cholesterol levels increase. The mitochondrial cholesterol accumulation determines an increase in ROS-induced oxidative stress.

Niemann–Pick Disease (NPD) is a rare autosomal recessive lysosomal storage disorder. NPD comprises three different pathologies, namely NPD type A, B, and C. NPD type A and B are now most commonly categorized as acid sphingomyelinase deficiencies (ASMDs), as they are caused by mutations in the gene SMPD1, which codes for sphingomyelin phosphodiesterase 1. However, NPD type C is a neurological disorder caused by mutations in the NPC1 or NPC2 genes, leading to defects in cholesterol trafficking and esterification. In ASMD types A and B, defects in sphingomyelin phosphodiesterase 1 result in a lack of acid sphingomyelinase activity, leading to the accumulation of sphingomyelin, cholesterol, and other lipids in lysosomes (Figure 1). Lung disease is common in ASMD type B, although pulmonary involvement may arise in all forms of the disease. Today, there are only limited therapeutic options available to treat NPD. This review aims to describe the state of the art on the genetic basis and lung involvement in ASMD types A and B, and NPD type C. It also discusses other clinical manifestations and the available therapeutic options (Table 1) [1,2].

## 2. Materials and Methods

A non-systematic, narrative literature review was conducted. The PubMed and Scopus search engines were used to collect the relevant articles from the international literature (last access to search engines: 1 December 2023). The search strategy included combinations of the following keywords and was limited to English-written articles only: Niemann–Pick Disease, Niemann–Pick Disease type A, Niemann–Pick Disease type B, Niemann–Pick Disease type A/B, Niemann–Pick Disease type C, acid sphingomyelinase deficiency, ASM, ASMD, *SMPD1*, *NPC1*, *NPC2*, Olipudase α, miglustat, lung transplant, therapy, enzyme replacement therapy, radiology, clinics, Niemann–Pick cells. Additional eligible studies were retrieved from bibliographies of the most cited studies and book chapters. Two authors independently screened abstracts from all the collected articles; in the case of a contrasting opinion on an article, a third author screened and decided whether to include it for the analysis. Full texts of the included articles were examined and relevant data summarized.

## 3. The Genetic Basis of Niemann–Pick Disease Type A and B: Disease-Causing Mutations and Genotype–Phenotype Relationships

ASMD is a group of inherited metabolic disorders caused by genetic mutations that affect the processing and transport of lipids, especially sphingomyelin and cholesterol, within cells. These mutations disrupt the function of specific enzymes or proteins involved in lipid metabolism, resulting in the abnormal accumulation of lipids in various tissues and organs.

The genetics of Niemann–Pick Disease are complex and involve several types, including types A, B, C1, and C2, each with distinct genetic causes. NPD types A and B (ASMD) are caused by mutations in the SMPD1 gene, which is located on 11p15.1-p15.4. This gene provides instructions for producing an enzyme called acid sphingomyelinase (ASM), which is responsible for breaking down sphingomyelin into ceramide and phosphocholine. While ASMD type A is always inherited as an autosomal recessive disease, ASMD type B is not only autosomal recessive. In some cases, individuals with one normal copy of the gene and one mutated copy (carriers) can have mild symptoms. Takahashi et al. emphasized the connection between specific mutations in the SMPD1 gene and the resulting clinical manifestations in Niemann–Pick Disease [3].

In ASMD type B, although the typical mode of inheritance is autosomal recessive, there can be cases where individuals who are carriers (heterozygous for the mutation) may exhibit mild symptoms or have some manifestations of the disease. The severity of symptoms in carriers can vary, and this phenomenon is often referred to as “carrier or heterozygote manifestations”. It means that, in some genetic disorders, individuals with only one copy of the mutated gene may show milder forms of the condition or may have subtle symptoms [4].

Small deletions or nonsense mutations that result in a truncated ASM polypeptide, as well as missense mutations that render the enzyme noncatalytic, are characteristic of ASMD type A. These mutations result in a severely deficient or non-functional ASM enzyme. As a result, sphingomyelin cannot be properly metabolized, leading to the buildup of sphingomyelin in cells. The accumulation of this substance is responsible for the severe neurovisceral symptoms observed in ASMD type A. ASMD type B is primarily caused by missense mutations that result in a defective enzyme with residual catalytic activity. Although the enzyme is defective, it still retains partial functionality, enabling the partial breakdown of sphingomyelin. This residual activity is associated with a milder non-neuronopathic type B phenotype, which typically presents with hepatosplenomegaly and lung involvement but lacks severe neurological symptoms seen in ASMD type A. Recent developments in molecular biology have identified more than 100 mutations in *SMPD1* in patients with ASMD type A/B, which are listed in the Human Gene Mutation Database (HGMD). Most of these mutations are missense (65.4%) or frameshift (19%) mutations. A deletion mutation, NM_000543.4(SMPD1):c.1829_1831delGCC (p.Arg610del), was considered the most frequent mutation reported worldwide. This mutation was associated with an attenuated ASMD type B phenotype [5]. Forty mutations were studied in vitro, and their impact on ASM activity was assessed. Twelve of these mutations retained some enzymatic activity, with eleven of them found in patients with ASMD type B. One specific mutation, p.Phe572Leu, retained 30% activity and was identified in a patient with ASMD type A, along with another mutation, p.Gly247Ser [6,7]. The study also noted six mutations associated with type A disease, three of which were found in Jewish Ashkenazi patients, where the disease is relatively common [8,9,10,11]. Moreover, Gluck and colleagues investigated 12 Arab Israeli families with ASMD type A in the lower Galilee and the West Bank, Palestine. Through a molecular analysis, they discovered a novel genetic mutation, a single base pair deletion in the *SMPD1* gene known as 677delT, which likely contributes to the development of ASMD type A in these patients.

## 4. Acid Sphingomyelinase Deficiencies: Clinical Aspects in Niemann–Pick Disease Type A and B

ASMD (NPD type A and B) are autosomal recessive lysosomal storage disorders caused by a deficiency in the activity of acid sphingomyelinase (ASM). This enzyme regulates the homeostasis of lysosomal sphingomyelin (SM), and its inactivation leads to the accumulation of SM, cholesterol, and other lipids in lysosomes [12]. ASM is encoded by the SMPD1 (sphingomyelin phosphodiesterase 1) gene, which is located on chromosome 11. More than 180 pathogenic mutations, including missense and nonsense mutations, deletions, and splicing abnormalities, have been described in the SMPD1 gene in patients with ASMD type A and B [13]. ASMD type B is a pan-ethnic disorder found worldwide. The majority of cases are located in the United States (USA), western Europe, Saudi Arabia, Turkey, and Tunisia. ASMD type A is more common in individuals of Ashkenazi Jewish descent than in the general population [14]. The difference between ASMD type A and B is based on the activity of ASM. In type A, there is a significant reduction in ASM activity (<5%), while in type B, there is a higher residual activity of the enzyme [12]. The accumulation of sphingomyelin and other lipids in cells such as hepatocytes, monocytes, and macrophages may lead to organ damage and dysfunction. At lung histology, the so-called vacuolated foamy macrophages, known as “Niemann–Pick cells”, can be found in the alveolar spaces, alveolar septa, and pleura. Niemann–Pick cells typically exhibit intense blue staining with the May Grunwald–Giemsa and the Schmorl reaction, and they are also referred to as “sea-blue histiocytes”. These foam cells accumulate in various organs, such as the liver, spleen, lungs, cerebral and cerebellar cortices, and bone marrow, and they can lead to organ failure. The severity of clinical symptoms is associated with the level of remaining acid sphingomyelinase activity [15]. Since foam cells can accumulate in the lungs, bronchoalveolar lavage (BAL) is a useful diagnostic tool in the work-up of ASMD. It is capable of detecting foam cells, although its use should be carefully considered due to the invasive nature of the procedure [16].

### 4.1. Niemann–Pick Disease Type A (ASMD Type A)

NPD type A is the most severe form. It is a neurodegenerative disease leading to death within the first years of life in most cases. NPD type A is characterized by progressive neuro- and psychomotor degeneration with gradual worsening of hypotonia, leading to a loss of language and mobility. The disease affects the cerebellum and cerebrum. A severe myelin deficiency and accumulation of foamy cells and lipid-laden glial cells can be retrieved in brain specimens from infants with NPD type A [13,15]. The first manifestation in NPD type A is organomegaly. A study conducted on ten patients affected by NPD type A showed hepatosplenomegaly was present by 4 months of age in association with abnormally elevated levels of liver enzymes, and total and direct bilirubin. Neurological symptoms more often begin by 9 months. Children have a sleep disorder with frequent arousals accompanied by periods of crying for hours. A growth delay and progressive hypotonia with a loss of the deep tendon reflexes subsequently arise accompanied by a progressive reduction in infant sucking with an insufficient intake of calories. In all the patients, the neurodegeneration causes frequent respiratory infections mostly related to aspiration [17]. In NPD type A, macular cherry-red spots are detectable in all infants by 12 months. This term describes the ophthalmoscopic appearance of the retina in neurometabolic disorders, like Niemann–Pick Disease. The red color of the fovea (in Caucasian patients) contrasts with the surrounding retina, which is pale for the accumulation of lipids and sphingolipids in the ganglionic cells of the retina at the macula [18,19].

### 4.2. Niemann–Pick Disease Type B (ASMD Type B)

NPD type B is a multisystemic disease with phenotypic heterogeneity and a variable severity and progression rate between patients. The most common clinical manifestations at onset are represented by splenomegaly and hepatomegaly. Splenomegaly is associated with recurrent bleedings, like recurrent epistaxis. However, there is no clear correlation between the rate and severity of bleeding and platelet counts, a characteristic manifestation of the disease [20]. Hepatomegaly is caused by the accumulation of the lipid-laden macrophages in the reticuloendothelial system of the liver. It can cause liver dysfunction, leading to cirrhosis and hepatic failure in some cases. Hepatic involvement, together with lung involvement, represents the most common cause of death in NPD type B [15,21]. The elevated levels of cholesterol, triglycerides, and LDL and very low levels of HDL cause, in children and adults, atherosclerosis with arteries’ calcification. Patients with ASMD type B may suffer from cardiac disease, and almost 10% have valve or coronary disease [15,21]. Skeletal involvement is also frequent in NPD type B. Delayed skeletal maturation with a growth restriction and short stature is typical. Osteopenia and osteoporosis with fractures may then occur. Moreover, Niemann–Pick cells can infiltrate the reticuloendothelial system of the bone marrow, causing leukopenia and thrombocytopenia [21,22].

### 4.3. Niemann–Pick Disease Type A/B (ASMD Type A/B)

An intermediate form of the disease, called ASMD or Niemann–Pick type A/B, has been described. Patients usually show all the symptoms of NPD type B but there is also neurological involvement, with extrapyramidal and cerebellar signs, nystagmus, mental retardation, and psychiatric disorders. The most common neurologic abnormalities are mild hypotonia and hyporeflexia. The onset of neurologic symptoms is later than in patients with NPD type A and are typically not characterized by a rapid progression. The precise mechanisms at the basis of the neurological involvement are not known yet. Indeed, in anatomopathological studies, the brain of patients affected by NPD type A/B with neurological involvement does not present evident abnormalities, due to a residual sphingomyelinase activity. Furthermore, one third of patients may have retinal involvement, although not always associated with the development of neurological deficits. It has also been reported that homozygotes and heterozygotes for the mutation ΔR608 seem to have a neuroprotective effect, and macular halos and cherry-red spots are less frequent in patients with this genotype [15,23,24,25].

## 5. Lung Involvement in ASMD

Respiratory complications may occur in all three types of Niemann–Pick Disease, with type B being the most common. Pulmonary complications are uncommon in NPD type A, and they usually consist of recurrent respiratory infections, interstitial lung disease, and aspiration pneumonia [1]. Patients with NPD type A typically succumb to respiratory failure by the age of 3. In contrast, patients with NPD type B exhibit a more diverse clinical phenotype and life expectancy.

Infants with NPD type B can develop progressive respiratory symptoms and often experience frequent respiratory infections due to aspiration, which can ultimately lead to severe respiratory failure [6]. In type B disease, patients more frequently reach adulthood, exhibiting a highly variable disease course with a wide range of clinical manifestations, from asymptomatic cases to severe respiratory failure. When symptoms are present, they include a recurrent cough, moderate exertional dyspnea, and frequent respiratory infections. Notably, there have been rare cases of rapidly fatal lung disease reported. In these patients, progressive pulmonary disease led to exertional dyspnea, ultimately resulting in respiratory failure and dependence on high-flow oxygen [1].

Specific lung manifestations have been described in NPD type B, including interstitial lung disease (ILD), pulmonary hypertension, alveolar hypoventilation, upper airway obstruction (with or without obstructive sleep apnea syndrome), and recurrent airway infections caused by mucous membrane swelling [26].

The common pathogenetic process involves the accumulation of Niemann–Pick cells in the alveolar septa, bronchial walls, and pleura, leading to a progressively restrictive pattern that can be detected with pulmonary function tests [1]. Radiology can aid in diagnosing pulmonary involvement in NPD type B. High-resolution computed tomography (HRCT) can reveal widespread lung abnormalities.

HRCT scans reveal the presence of predominantly basal interstitial lung disease (ILD) in the vast majority of cases. This is characterized by thickened interlobular septa, interlobular lines, and ground-glass opacities. Ground-glass opacities may be more noticeable in the upper lung zones, often caused by the partial filling of alveoli with Niemann–Pick cells. In contrast, interlobular septal thickening is often more pronounced in the lower lung zones. Additionally, peri-bronchovascular interstitial thickening may be observed along with interlobular septal thickening. Centrilobular nodular opacities or a “crazy paving” pattern, with the typical presence of ground-glass opacities and thickening of interlobular septa, may also be observed and mimic lipoid pneumonia [1,12,27]. Other non-specific HRCT findings, such as segmental atelectasis and bronchiectasis, have been reported and have been specifically correlated with a particular SMPD1 gene mutation (p.Ala461⁄4) [2]. In one case report, focal areas of low attenuation, resembling cysts, were identified alongside ground-glass opacities, primarily in the lower lobes of the lungs [1,12]. It is important to note that the HRCT lung involvement is not a reliable predictor of lung function in these patients [28]. Bronchoscopy can detect the presence of Niemann–Pick cells or “sea-blue histiocytes” in the bronchoalveolar lavage fluid and lung biopsy specimens. These cells are large, multivacuolated histiocytes containing fine and coarse granules that stain deep blue with the May Grunwald–Giemsa stain. Moreover, bronchoalveolar lavage fluid may show inflammatory cells, suggesting the presence of a concomitant inflammatory process within the alveolar space, which may contribute to the respiratory symptoms observed in these patients [1]. Patients with NPD type B often suffer from pulmonary hypertension, which can be confirmed using echocardiography and catheter angiography. Multiple pulmonary artero-venous fistulas can be detected [1]. Pulmonary function tests usually show normal lung volumes with a reduced diffusion capacity for carbon monoxide (DLCO). Even in cases of advanced Niemann–Pick Disease with interstitial infiltrates and a severely impaired DLCO, lung volumes and flow rates may remain relatively preserved [1]. Notably, some of the pulmonary symptoms may be related to a restrictive pattern due to the severe hepatosplenomegaly observed in certain patients. Hepatosplenomegaly can indeed reduce the diaphragm excursions, and induce a diaphragm elevation, which determines a restriction of the lung volumes [2]. In conclusion, lung involvement in NPDB exhibits high heterogeneity, featuring clinical and radiological manifestations of varying severity. Differential diagnoses can be challenging due to the absence of specific clinical symptoms and imaging findings [2]. The main characteristics of lung involvement in NPD type A and B are summarized in Table 2.

## 6. Therapeutic Strategies in ASMD

Therapeutic strategies in NPD type A and B are still limited and often associated with only mild improvement in the prognosis and quality of life. Recently, Geberhiwot et al. [29] published the first consensus guidelines on clinical management of these rare diseases. Therapeutic options include enzyme replacement therapy, solid organ transplantation (liver, lung), bone marrow transplantation, gene therapy, symptomatic therapies. Table 3 summarizes the main therapeutic options.

### 6.1. Enzyme Replacement Therapy

Enzyme replacement therapy (ERT) is the best therapeutic option in NPD and is considered a disease-modifying therapy, able to guarantee the widespread enzyme delivery to the affected organs. Olipudase α, a human recombinant acid sphingomyelinase, is the first approved ERT for the long-term treatment of ASMD manifestations other than those involving the central nervous system (CNS). Olipudase α is an intravenous treatment geared to treating the systemic manifestations of the disease. The drug is capable of replacing defective or deficient ASM. Biweekly infusions of the recombinant enzyme are required for the long-term treatment. Four clinical trials have been performed to test Olipudase α. The first trial was a single-center, open-label, single-ascending-dose trial performed on 11 adult patients suffering from NPD type B. This trial established that the safest maximum starting dose was 0.6 mg/kg in humans, with the possibility to escalate [30]. Subsequently, a safety and tolerability trial was conducted on five adult patients with ASMD, to whom escalating doses of Olipudase α were administered every 14 days for 26 weeks [31]. These studies allowed the realization of the ASCEND study, a phase II/III, international, multicenter, randomized, double-blind, placebo-controlled efficacy trial in which 36 adult patients with ASMD were randomized to receive intravenously Olipudase α or a placebo [32]. The study showed that Olipudase α may significantly improve diffusing capacity of the lung for carbon monoxide (DLCO) and reduced the spleen and liver volumes compared to the placebo. Finally, a phase I/II, international, multicenter, open-label trial (ASCEND-Peds) tested Olipudase α in 20 pediatric patients reaching the primary safety endpoint and an improvement in hepatomegaly, splenomegaly, and DLCO [33]. 

According to the 2023 consensus guidelines [29], strict monitoring should be performed during the long-term treatment with Olipudase α, particularly through the evaluation of the improvement in lung function, the reduction in liver and spleen volume, the adequate growth in pediatric patients, the maintenance of an adequate lipid profile. 

### 6.2. Solid Organ Transplantation

In the literature, a significant number of authors have described the transplantation of affected solid organs as possible treatment options in ASMD. However, it is worth noting that since ASMD affects all the cells of the body, the transplantation of a damaged organ does not prevent the recurrence of the disease in the donor organ once transplanted or the progression of the disease in the other organs of an affected patient.

#### 6.2.1. Lung Transplantation

Patients suffering from NPD type A and B are at an increased risk of respiratory infections, aspiration pneumonia, and, especially for adult patients with NPD type B, progressive interstitial lung disease. Lung transplantation may represent a feasible therapeutic option in NPD type B with an end-stage lung disease [2]. To date, five cases of lung transplantation have been reported with variable allograft outcomes. Mannem et al. described a double-lung transplant in a 62-year-old man who rapidly died of septic shock and NPD type B pulmonary recurrence. O’Neill et al. [34] reported a double-lung transplant for pulmonary fibrosis in a man diagnosed with NPD type B after splenectomy. He survived 267 days post-transplant. Ding et al. [35] described a double-lung transplantation in a man firstly diagnosed with NPDB after liver transplantation, who was alive 35 months post-transplantation without NPDB recurrence. Tirelli et al. [2] described a 34-year-old woman, the youngest patient to be transplanted for NPD type B with lung involvement, still living 3 years after the lung transplant. Finally, Mora et al. [36] reported a double-lung transplantation in a 57-year-old man with diffuse interstitial lung disease. The patient did not show recurrence of the disease nor relevant complications 23 months post-transplantation. 

A lung transplant should be considered a therapeutic option for severe lung impairment in NPD type B. In case of a suspected undiagnosed storage disorder, a post-transplant histopathological and genetic test for ASMD should be performed. 

#### 6.2.2. Liver Transplantation

Liver involvement is frequent in NPD type A and B. It is characterized by elevated transaminases, liver enlargement, fibrosis, and cirrhosis, especially in the third and fourth decade. When the liver becomes cirrhotic, other complications might manifest, as portal hypertension, ascites, hepatic encephalopathy, and esophageal varices. Hepatic failure is one of the leading mortality causes in NPD; thus, liver transplantation might be considered as a therapeutic option. It has been described in patients with NPD type B and hepatic dysfunction, with good outcomes not only in liver function but also in growth parameters and lung function [37,38].

### 6.3. Bone Marrow Transplantation

Bone marrow transplantation has been reported as a possible therapeutic strategy, with good results in terms of a reduction in hepatosplenomegaly, the correction of the metabolic defect, and improvement in blood counts [39]. Anyway, the possible complications secondary to the transplantation (i.e., graft versus host disease, renal failure, risk of infection due to immunosuppressive therapy) are limiting this therapeutic option [40]. Moreover, bone marrow transplantation does not improve nor stabilize neurologic involvement [41].

### 6.4. Gene Therapy and Other Innovative Approaches

Gene therapy has been tested to be safe and effective in the animal model of an Acid-Sphingomyelinase-Deficient Mouse (ASMKO mouse). Different approaches have been adopted for vehicle gene therapy: an adeno-associated virus (AAV) vector directed to the liver [42], a recombinant human ASM (rhASM) direct brain injection [43], bone marrow cells transduced with retroviral vectors encoding human ASM [44]. Efficacy and safety of gene therapy in humans need to be demonstrated with targeted future clinical trials.

Another innovative approach is based on molecular chaperone therapy, and particularly Hsp70 proved to be able to stabilize lysosomes, activate ASM, and stabilize the infused recombinant enzymes, as reported by Kirkegaard et al. In their work, they reported that the reduced ASM activity in cells from patients affected by NPD type A and B is associated with lysosomal instability. Recombinant Hsp70 proved to be able to correct this phenotype. This result might open the door for new treatments with compounds that are able to enter the lysosomal lumen via the endocytic delivery pathway [45]. 

### 6.5. Symptomatic Therapies

Splenomegaly can lead to hypersplenism. Total splenectomy should be avoided or performed only in selected cases, when other less invasive and safer treatment options have failed or cannot be performed. The partial embolization of the splenic artery or partial splenectomy are the most viable options. In the event of partial or total splenectomy, patients should receive vaccinations for Streptococcus pneumoniae, Neisseria meningitidis, and Hemophilus influenzae type B. It is worth noting that splenectomy may worsen lung and liver function due to the increased accumulation of sphingomyelin in these organs [29]. 

Respiratory system involvement might lead to respiratory failure, even in young patients. Therefore, long-term oxygen therapy and non-invasive ventilation may be necessary. Anecdotally, there have been reports of whole lung lavage. Vaccination against common respiratory pathogens should be performed [46].

Dysphagia should be carefully investigated because it is associated with an increased risk of aspiration pneumonia. Speech and language therapy might be beneficial. In certain cases, percutaneous endoscopic gastrostomy or nasogastric tube feeding can be used to ensure proper caloric intake and reduce the risk of aspiration.

Abnormal linear growth with delayed skeletal maturation has been described as quite common in young patients, and there are some reports in the literature on the use of a growth hormone to treat this condition [47].

Thrombocytopenia is quite common. As a result, nosebleeds are common but often resolve on their own. Attention should be paid to esophageal varices secondary to liver disease, as life-threatening hemorrhages can occur. Hematologist consultation is recommended for the proper management of bleeding disorders [48].

Finally, anxiety, depression, sleep disturbances, fatigue, and chronic pain are other relevant manifestations that must be carefully considered and treated. However, there is currently no consensus on the best strategy [49].

## 7. The Genetic Basis of Niemann–Pick Disease Type C: Disease-Causing Mutations and Genotype–Phenotype Relationships

Niemann–Pick Disease type C is a rare autosomal recessive genetic disorder characterized by progressive neurodegeneration. Approximately 95% of cases are caused by mutations in the *NPC1* gene (NPD type C1); 5% are caused by mutations in *NPC2* (NPD type C2). The clinical manifestations of types C1 and C2 are similar because the respective genes are both involved in egress of lipids, particularly cholesterol, from late endosomes or lysosomes [50]. 

More in detail, *NPC1* and *NPC2* genes encode for proteins that play a crucial role in the intracellular transport of lipids, particularly cholesterol. Mutations in *NPC1* or *NPC2* genes lead to a shortage of functional protein, which impairs the movement of cholesterol and other lipids within cells. As a result, these lipids accumulate in cells, disrupting normal cellular functions that rely on lipids, such as the formation of cell membranes. The accumulation of lipids, along with impaired cellular functions, ultimately leads to cell death. This cell death results in the tissue and organ damage characteristic of Niemann–Pick Disease types C1 and C2. The accumulation of lipids in cells due to NPC mutations has broad implications for various organs and tissues, leading to the wide range of symptoms observed in individuals with Niemann–Pick Disease type C. These symptoms can include neurological problems, liver and spleen enlargement, and difficulties with movement and coordination, among others [51]. 

The *NPC1* gene, in particular, exhibits significant genetic variability, with around 300 identified disease-causing mutations and over 60 polymorphisms described. Of interest, the G3097>T transversion in *NPC1* is the basis for so-called Niemann–Pick D (Nova Scotian variant) [52]. In the past, early studies suggested that approximately 95% of affected families had mutations in the *NPC1* gene. However, more recent research has identified a small number of families with mutations in the *NPC2* gene, although these cases remain rare [53]. The *NPC1* gene, located on chromosome 18q11-q12, consists of 25 exons and spans 56 kb. One of the prominent mutations in the NPC1 gene is the p.I1061T allele [54]. This mutation is particularly common, accounting for approximately 20–25% of alleles in patients diagnosed in France and the United Kingdom. It is also prevalent in a specific Spanish American isolate from the upper Rio Grande valley, but much less frequent in Portugal, Spain, or Italy. When present in the homoallelic state (meaning both alleles carry the mutation), it leads to significant abnormalities in cellular cholesterol trafficking in patient fibroblasts. Interestingly, it is associated with a juvenile neurologic onset form of the disease [55]. In the heteroallelic state (when paired with another mutation), it has not been found to be associated with the most severe infantile neurologic onset form of the disease. The p.I1061T mutant protein is functionally defective, leading to protein misfolding and its selection for degradation within the endoplasmic reticulum. This characteristic makes it a potential target for chaperone therapy, a promising approach for treating certain genetic disorders by assisting the correct folding of mutant proteins. The specific type and location of mutations in the NPC1 gene can significantly influence the severity of NPC1 in affected individuals. Mutations that disrupt critical functional domains or result in a complete loss of functional protein tend to be associated with more severe forms of the disease. In particular, patients with NPC1 who have nonsense or frameshift mutations generally experience a more severe neurological course. These types of mutations often result in a nonfunctional or truncated NPC1 protein, leading to a more severe phenotype. In patients with NPC1, missense mutations have highlighted the functional importance of specific domains within the NPC1 protein. Homozygous mutations in the sterol-sensing domain of the NPC1 protein are particularly harmful. These mutations lead to the absence of mature NPC1 protein. Patients with these mutations exhibit a very severe disease phenotype, both biochemically and clinically. The cysteine-rich luminal loop of the NPC1 protein seems to be a hotspot for genetic mutations associated with Niemann–Pick Disease type C1. This region contains about one-third of all described mutations in the NPC1 gene. Despite being a common site for mutations, the impact of these mutations can vary significantly both at the cellular and clinical levels [56,57].

Of particular note is that the cysteine-rich luminal loop contains the three most frequent mutations discussed earlier. Additionally, mutations found in this loop tend to result in a less severe impairment of cellular trafficking, leading to what is referred to as a “variant” phenotype. This means that mutations in this region may cause a milder form of the disease, affecting both the cellular processes and clinical manifestations to a lesser extent compared to mutations in other regions of the NPC1 gene [58,59].

Understanding the specific location and nature of mutations within the *NPC1* gene, especially in regions like the cysteine-rich luminal loop, is crucial for predicting the clinical course of NPC1 in affected individuals. Variability in the impact of these mutations underscores the complexity of the disease and highlights the need for a detailed genetic analysis in diagnosing and understanding NPC1 and related disorders.

The *NPC2* gene, also known as HE1 (Human Epididymis Protein 1), is mapped to the long arm of chromosome 14 at position 14q24.3 [60]. A specific mutation, denoted as E20X, is relatively frequent in individuals with NPD type C2 and results in a truncated NPC2 protein, which can lead to severe clinical phenotypes [53,61]. Many mutations in the *NPC2* gene cause the resulting protein to be truncated, preventing it from functioning properly. These mutations are associated with very severe clinical phenotypes. Severe truncating mutations often result in early-onset, rapidly progressing forms of the disease. Unlike truncating mutations, missense mutations may not completely abolish protein function, allowing for a more varied range of phenotypes. Individuals with missense mutations may exhibit a spectrum of clinical manifestations, including juvenile and adult-onset forms of the disease.

In both NPD type C1 and C2, the study of a large number of families has demonstrated that mutations in these genes correlate with the neurological form of the disease, while the correlation with the systemic manifestations needs to be better clarified.

## 8. Niemann–Pick Disease Type C: Clinical Aspects

Niemann–Pick Disease type C (NPD type C) is a rare autosomal recessive lysosomal storage disorder caused by defects in cholesterol trafficking and its esterification. The prevalence of the disease ranges between 1 in 120,000 and 1 in 150,000 [62].

The clinical presentation of NPD type C is extremely diverse. Although once considered rare, it is now recognized that NPD type C is more prevalent than previously thought and should be more accurately considered as an under-recognized disease, particularly in adults [63]. Indeed, the lack of a defined phenotypic spectrum makes a clinical diagnosis challenging. The age of onset varies from the perinatal period to adulthood. Affected patients can live up to 70 years [63]. NPD type C is a neurovisceral condition. The heterogeneous organ involvement typically occurs at different times, follows an independent course, and usually precedes the onset of neurological symptoms. At the time of diagnosis, systemic involvement is not present in 15% of pediatric patients and in almost 50% of adult-onset patients. NPD type C is typically characterized by cerebellar ataxia, dysarthria, dysphagia, and progressive dementia. Most cases also exhibit a characteristic vertical supranuclear gaze palsy. Other common symptoms include seizures, cataplexy, narcolepsy, and dystonia. Furthermore, adult-onset NPD type C can be misdiagnosed because its clinical features overlap with those of more common hereditary ataxic disorders [64]. The majority of patients, including adults, die from progressive and fatal neurological diseases, while only a small subset of adult patients die from hepatic or respiratory failure. Except for the infantile forms, the systemic disease is usually not very severe, and fatal lung involvement has been reported in only a few patients with NPC2 mutations [63,65,66,67]. Thrombocytopenia and leukopenia may occur due to bone marrow infiltration by Niemann–Pick cells, while hepatosplenomegaly is a common manifestation of systemic involvement [68].

Four phenotypes of NPD type C have been described. All of these conditions are characterized by systemic involvement, followed by neurological symptoms.

*Fetal and early infantile form:* Fetal hydrops and fetal ascites are the most common clinical presentations of the disease during the perinatal period, which could rapidly lead to death. About 40% of patients present with prolonged neonatal cholestatic jaundice and progressive hepatosplenomegaly. It usually regresses spontaneously; in only 10% of cases, it worsens and leads to liver failure. In some cases, severe respiratory failure, including fatal cases, may occur. This phase could be followed by progressive neurodegeneration, ultimately leading to death within the first few years of life. In early childhood, the first neurological symptoms usually include central hypotonia and delayed developmental motor milestones, followed by the loss of acquired motor skills, mental regression, and spasticity with pyramidal tract involvement. An intention tremor is common, while seizures are typically absent. Brain imaging reveals indications of leukodystrophy and cerebral atrophy. The prognosis is rarely more than 5 years.

*Late infantile form:* This form presents with the development of seizures, a progressive language delay, gait problems, ataxia, hearing loss, vertical supranuclear gaze palsy, and eventually cataplexy and dementia. Death most commonly occurs between the ages of 7 and 12.

*Juvenile form:* This is the most common form, occurring in mid-to-late childhood. It is characterized by the emergence of school difficulties, ataxia, and a loss of motor abilities, followed by seizures and dementia. In children aged 5–12 years, moderate splenomegaly is common. The lifespan ranges from the late teens to 30 years old or beyond.

*Adult-onset form:* In adolescent and adult patients, the disease often manifests with a more gradual and subtle onset. In one-third of cases, patients exhibit only a psychiatric presentation (typically psychosis) with a normal neurological examination. Some patients exhibit a milder form of juvenile-onset ataxia, dystonia, and dysarthria with varying degrees of cognitive dysfunction. Movement disorders are very common (58%), while epilepsy is rare (15%). Dementia can often manifest [62].

The diagnosis of NPD type C is challenging. Biochemically, the diagnosis could be confirmed through filipin staining, which demonstrates free cholesterol storage in fibroblasts. However, nowadays, the diagnosis is primarily based on genetics, which involves identifying specific mutations in the NPC1 and NPC2 genes. NPC1 encodes NPC protein 1, a transmembrane protein, while NPC2 encodes NPC protein 2, a soluble lysosomal protein involved in intracellular cholesterol trafficking. In the event that NPC protein 1 or 2 is dysfunctional, cholesterol tends to accumulate in the intracellular lamellar bodies of alveolar macrophages and alveolar type 2 cells, compromising surfactant production and homeostasis [55].

Lung involvement has been reported, particularly in patients with NPC2 mutations, leading to clinical manifestations such as alveolar proteinosis [69].

### 8.1. Imaging Techniques in the Diagnosis and Follow-Up of NPD Type C

Radiological examinations in NPD type C do not show specific findings.

#### 8.1.1. Gastroenterological Tract

Because of the common involvement of the liver and spleen, abdominal ultrasound with volumetry can be used in routine clinical practice to assess the size of these organs. Moreover, a quantitative measurement of liver and spleen volume can be assessed with magnetic resonance imaging (MRI) or a CT scan [70]. 

#### 8.1.2. Neurological Tract

Although a correlation between neuro-imaging (a potential biomarker) and clinical neurologic manifestations has not been already demonstrated, several studies have focused on this topic. NPDs are characterized by a significant hypometabolism in different brain areas: the left anterior cingulate cortex and bilateral posterior cingulate cortex, bilateral cerebellum, basal ganglia, left parietal cortex, bilateral temporal cortex, bilateral insular cortex, bilateral midbrain, tegmentum, and bilateral postcentral cortex. In the clinical setting, magnetic resonance imaging (MRI) is actually considered useful for identifying these abnormalities. Some studies demonstrated that 18-FDG-PET (positron emission tomography) and single-photon emission computed tomography (SPECT) can also be useful in clinical practice [71].

#### 8.1.3. Pulmonary Tract

HRCT is the most useful tool to assess lung involvement. In NPD type C, signs of lung involvement consist of ground-glass opacities, a reticular or reticulonodular pattern, with or without septal lines, and eventually honeycombing. These alterations mostly involve the lower lung zones, which can later progress to the entire lung [72,73]. Moreover, a crazy paving pattern, cysts, or pulmonary centrilobular nodular opacities can also be present [74]. Nonspecific rare HRCT findings are represented by segmental atelectasis and bronchiectasis [74].

## 9. The Lung Involvement in Niemann–Pick Disease Type C

Lung involvement in NPD type C is closely related to an imbalance in the intracellular trafficking of cholesterol, especially in alveolar epithelial cells type II. These cells are responsible for the production, storage, and release of the surfactant [75,76].

The Niemann–Pick Disease type C pathway consists of two essential components: NPC protein 1, a large transmembrane protein, and NPC protein 2, a soluble lysosomal protein. These proteins regulate the intracellular transportation of cholesterol. Any interruption or obstruction in this pathway leads to the buildup of cholesterol in alveolar type II cells and alveolar macrophages [58].

Liu et al. have provided insights into the impact of impaired NPC1 function. Mice lacking functional NPC1 genes showed significant macrophage infiltration and cholesterol accumulation in their lungs, providing insight into the potential effects of disrupted cholesterol trafficking in the respiratory system [77,78,79,80].

Progressive lung involvement has been reported in a few patients with Niemann–Pick Disease type C, with the majority of cases observed in patients with NPC2. In these cases, progressive lung disease is not caused by the harmful effects of aspiration pneumonia. To date, only a few cases of lung involvement have been described in patients with NPC1, and this involvement has not been found to be secondary to aspiration. One documented case involved a 16-year-old patient with NPC1, who experienced a persistent cough and excessive bronchial secretions [81,82].

The NPC pathway has been studied in the lungs of mice (Roszell et al.). By blocking this pathway, NPC1 protein was observed to be concentrated on the limiting membrane of the lamellar bodies, while NPC2 was present in the lumen of these organelles and in the pulmonary lavage fluid [83,84]. These findings provide a potential explanation for the improvements observed by Palmeri et al. in a patient with NPC1 following bronchoalveolar lavage. They may also illuminate the development of interstitial lung disease, especially in more severe neonatal/infantile forms of the disease compared to the more prevalent adult form, which mainly manifests as neurological deterioration.

The lung biopsy and lavage of patients with Niemann–Pick Disease type C also revealed the presence of foamy macrophages [85].

Staretz-Chacham et al. studied the pulmonary involvement in a group of 12 patients who were homozygous for the same NPC1 gene mutation (p.R404Q). Typical presentation signs included tachypnea and crackles upon auscultation, followed by prolonged periods of hypoxemia. X-ray and CT findings revealed bilateral enlarged alveolar infiltrates at the lung bases, with extensive interstitial involvement. BAL was positive for foamy macrophages containing numerous polymorphous cytoplasmic inclusion bodies. On post-mortem examination, the patient’s lungs exhibited an extensive infiltration of the alveoli and interlobular septa with large foamy macrophages and chronic inflammation, and no presence of a hyaline membrane or lung fibrosis [72,86,87].

While some individuals with this mutation develop neurological issues in adulthood, the distinctive pulmonary manifestations can be attributed to the specific p.R404Q mutation. This mutation disrupts the binding of the NPC1 protein to cholesterol-enriched NPC2, leading to the accumulation of cholesterol and pulmonary symptoms similar to those observed in individuals with NPC2 gene mutations [69,86,88].

## 10. Therapeutic Strategies in Niemann–Pick Disease Type C

A multidisciplinary approach for the treatment of Niemann–Pick Disease type C is recommended. Particularly, symptomatic therapies and speech and developmental therapies together with physical activity should be recommended in order to counteract neurodegeneration. They are currently the mainstream of treatment and can positively impact on the quality of life. Only one disease-modifying agent is available and approved in the majority of countries. The therapeutic strategies (available and still experimental) are described in Table 4.

### 10.1. Disease-Modifying Therapy

Today, only one disease-modifying drug, namely miglustat, is approved for the treatment of NPD type C. In particular, miglustat has been approved in the European Union, Canada, and Japan (but not by FDA in the United States of America) for treating progressive neurological complications in NPD type C.

Miglustat is an iminosugar, which acts as a substrate reduction treatment. It inhibits glucosylceramide synthase (GCS), an enzyme that catalyzes the first steps of glycosphingolipid synthesis, reducing their accumulation within lysosomes. Furthermore, it also serves as an inhibitor of non-lysosomal glucosylcerebrosidase (Gba2). It is the only disease-modifying treatment for NPD type C with neurological symptoms approved by regulatory authorities, except for the US Food and Drug Administration (FDA). The recommended dose for the treatment of NPD type C in adults and patients older than 12 years old is 200 mg three times a day. For children under 12 years of age, the dosage should be adjusted based on body surface area. The efficacy of miglustat lies in its mechanism of action: once having crossed the blood–brain barrier, it is able to directly target the lipid storage in the central nervous system [89,90]. Patients with NPC under therapy showed a significantly lower level of oxyesterol (in particular, cholestane-3β,5α,6β-triol concentrations) compared to untreated patients [91]. Since NPD type C can rapidly progress to neurodegenerative manifestations, miglustat should be introduced early after the diagnosis [63]. The first randomized clinical trial demonstrating the efficacy of miglustat in NPD type C was conducted in 2007 [92]. After that, several longitudinal cohort studies (lasting from 2 to 8 years) indicated a disease stabilization in terms of neurological involvement [90]. In these studies, it was observed that there was a greater efficacy in patients with juvenile and adult-onset phenotypes than those with infantile-onset (<6 years) forms [50]. Studies have also demonstrated an improvement in or at least stabilization of vertical supranuclear saccades palsy and gaze palsy in patients under treatment for 1 to 5 years [93,94]. A neuroprotective effect was also evaluated with studies of correlation between brain imaging and symptom severity [95] and with metabolic imaging studies, showing a reduction in neurodegeneration markers (choline, creatine, N-acetylaspartate, and their ratio) in treated patients [96,97,98,99]. In long-term cohort series, dysphagia, a risk factor for aspiration pneumonia and premature death, also improved or stabilized [100]. The most common side effect of miglustat is diarrhea, because the drug also inhibits intestinal disaccharidases, followed by a tremor and weight loss [101]. 

### 10.2. Symptomatic and Supportive Therapy

Patients with NPD type C should undergo regular functional assessment of the potentially involved organs so as to start adequate supportive and symptomatic therapy when needed [63]. 

Growth, mobility, strength, and balance should be regularly checked. Mobility aids and personalized rehabilitation programs can be helpful in training mobility and improving motility dysfunctions.

Speech and communication skills might be altered in NPD type C, so language therapy might be considered.

Spasticity is another frequent manifestation of the disease, which can be initially counteracted with non-pharmacological treatments, but often needs to be treated with pharmacological agents as well (i.e., Baclofen, Tizanidine, Benzodiazepines, botulinum toxin injections).

Bowel dysfunction is frequent, and strategies to modulate fecal consistency, including the use of laxatives, are helpful in avoiding fecal impaction. Parallelly, bladder dysfunction can also be present and urologist intervention is recommended in case of signs of a neurogenic bladder.

Postural drainage and drugs including Hyoscine hydrobromide (transdermal), Glycopyrronium (oral, subcutaneous, via gastrostomy), atropine (oral), or botulinum toxin injections should be considered when hypersalivation and consequent drooling are present.

Tricyclic agents (i.e., Protriptyline) and modafinil have proved to be efficacious agents in case of cataplexy. When epilepsy is diagnosed, this condition must be carefully evaluated by an expert neurologist, since many common anti-epileptic drugs (i.e., carbamazepine) might aggravate neurologic symptoms in NPD type C. 

Hearing devices should be considered in case of hearing loss. An annual hearing assessment is suggested in every patient with NPD type C.

Mental wellbeing and cognitive decline are two noteworthy aspects to pay attention to. Anxiety, depression, or psychosis and behavioral problems can be retrieved with a high prevalence in patients with NPD type C. The aid provided by a clinical psychology/psychiatric team could be helpful and, when needed, pharmacological treatment should be started. Cognitive decline can impact on quality of life, so strategies to help patients ensuring the safety in the living environment are essential.

### 10.3. Future Therapeutic Strategies

Other promising drugs are in the late stages of clinical trials, showing encouraging preliminary results. These include intravenous cyclodextrin and acetyl-L-leucine. Finally, gene therapy and stem cell treatments might represent future options, if validated in appropriate preclinical and clinical studies [90].

Numerous new disease-modifying agents are currently being studied, such as adrabetadex and arimoclomol, a heat-shock protein production stimulator. Despite promising preliminary findings in clinical trials, arimoclomol has not yet received marketing authorizations from the EMA and FDA. Additionally, trials for intrathecal adrabetadex have failed.

Other trials have been designed to test acetyl-L-leucine, an orally administered medication with both disease-modifying and symptomatic effects. Moreover, the drug may exert a synergistic mechanism with the already approved drug miglustat [102].

Finally, clinical trials are expected to determine whether intravenous cyclodextrin might be a viable therapeutic option.

If these new agents receive marketing approval, a combination therapy with miglustat may represent a new therapeutic approach, thanks to the observed synergistic mechanism of action. However, the specific combination and dosage need to be studied.

Finally, preclinical studies on gene therapy are currently underway. The transfer of specific genes could counteract lipid accumulation in affected organs. Infusing an adenovirus viral vector into the cerebrospinal fluid led to neurological and systemic improvement in a murine model of NPC1. However, the immunogenicity of viral vectors represents a limitation to their adoption. This obstacle should be carefully considered before starting clinical trials in humans [103].

## 11. Conclusions

Although ASMD (NPD type A and B) and NPD type C are rare diseases, significant efforts and progress have been made in recent years to elucidate their genetic basis and the biological mechanisms of the diseases, with the aim of developing effective treatment strategies. The identification of ASM deficiency in NPD types A and B, as well as defects in cholesterol esterification in NPD type C, has opened up new research opportunities. Two approved disease-modifying drugs are now available: ERT with Olipudase α for ASMD and miglustat for NPD type C. An early diagnosis and treatment are crucial for improving patients’ outcomes. Multidisciplinary discussion, as in ILD, is fundamental in the diagnostic work up [104]. Future research is necessary to gain a better understanding of the pathomechanism of these diseases and to explore new therapeutic options.

## Figures and Tables

**Table 1:** Main characteristics of ASMD (NPD type A and B) and NPD type C.

	ASMD Type A	ASMD Type B	NPD Type C
Age of onset	Childhood	Childhood and adulthood	Mainly childhood, adulthood (underecognized)
Lung involvement	Present	Frequent	Rare
Hepato-splenomegaly	Frequent	Frequent	Frequent
Liver disease	Present	Frequent	Present
Neurodegeneration	Frequent and rapidly progressive	Present and slowly progressive	Present and rapidly progressive (in younger patients)
Atherosclerosis	Present	Frequent	Not described
Growth delay	Frequent	Frequent	Not frequently described
Hypotonia	Frequent	Rare	Present

**Table 2:** Lung involvement in NPD type A, B, and C.

ASMD Type A	ASMD Type B	NPD Type C
Severity: high, death in childhood. Death often due to infections secondary to aspiration pneumonia	Severity: variable, from asymptomatic forms to death in adult age	Severity: progressive lung involvement (NPC2 > NPC1). In NPC1 death often for complications of aspiration pneumonia.
Symptoms: recurrent cough, dyspnea	Symptoms: recurrent cough, moderate exertional dyspnea	Symptoms: persistent cough, excessive bronchial secretions
Respiratory failure: occurs in childhood	Respiratory failure: occurs in adulthood	Respiratory failure: can occur in childhood
ILD: aspecific, few cases described only in childhood	ILD: predominantly basal ILD with thickened interlobular septa, interlobular lines, crazy paving pattern and ground-glass opacities (often present also in upper zones)	ILD: bilateral enlarged alveolar infiltrates at lung bases, with extensive interstitial involvement (*NPC1* mutation); alveolar proteinosis (*NPC2* mutation)
Pulmonary hypertension: not described	Pulmonary hypertension: recurrent (mainly linked to cirrhosis and back pressure)	Pulmonary hypertension: not described
Upper airways obstruction: not described	Upper airways obstruction: recurrent	Upper airways obstruction: not described
Respiratory infections: recurrent	Respiratory infections: frequent	Respiratory infections: frequent
Aspiration pneumonia: frequent	Aspiration pneumonia: frequent	Aspiration pneumonia: frequent (NPC1 > NPC2)
Bronchoscopy: not described	Bronchoscopy: Niemann Pick cells at BAL	Bronchoscopy: foamy macrophages with abundant polymorphous cytoplasmic inclusion bodies at BAL and lung biopsy
Pulmonary Function Tests: not described	Pulmonary Function Tests: normal lung volumes with reduced DLCO	Pulmonary Function Tests: not described

**Table 3:** Main therapeutic options in Acid Sphingomyelinase Deficiency (NPD type A and B).

Therapeutic Options in Acid Sphyngomyelinase Deficiency Type A and B	
Drug therapy	Enzyme replacement therapy: Human recombinant acid sphingomyelinase (Olipudase alpha)
Transplantation	Lung transplantation: few case reports in ASMD type B only
	Liver transplantation: few case reports in ASMD type B only
	Bone marrow transplantation: isolated case reports
Gene therapy (preclinical studies only)	Adeno associated viral vector, recombinant human ASM, bone marrow cells transduced with retroviral vectors encoding human ASM (Animal models only)
Symptomatic therapies	Splenomegaly: partial embolization of splenic artery or partial splenectomy
	Respiratory failure: long-term oxygen therapy and non-invasive ventilation
	Dysphagia and Aspiration risk: percutaneous endoscopic gastrostomy or nasogastric tube feeding
	Abnormal linear growth: growth hormone therapy in ASMD type B only
	Anxiety, depression, sleep disturbance, fatigue and chronic pain: no consensus on therapy

**Table 4:** Main therapeutic options (available and still experimental) in Niemann–Pick Disease type C.

Therapeutic Options in Niemann-Pick Disease Type C	
Drug therapy	Disease modifying therapy: Iminosugar (Miglustat)
Symptomatic therapies	Growth, mobility, strength and balance: mobility aids and personalized rehabilitation programs
	Speech and communication: language therapy
	Spasticity: non-pharmacological and pharmacological treatments (i.e., Baclofen, Tizanidine, Benzodiazepines, botulinum toxin injections).
	Bowel dysfunction: laxatives for modulating fecal consistency
	Neurogenic bladder: urologist intervention
	Hypersalivation: Postural drainage, Hyoscine hydrobromide (transdermal), Glycopyrronium (oral, subcutaneous, via gastrostomy), atropine (oral) or botulinum toxin injections
	Cataplexy: Tricyclic agents (Protriptyline) and modafinil
	Epilepsy: Expert neurologist consultation
	Hear loss: Hearing devices
	Anxiety, depression or psychosis and behavioural problems: Clinical psychology/psychiatric consultation and, when needed, pharmacological treatment
Future therapeutic strategies (gene therapy has preclinical studies only)	Intravenous cyclodextrin, adrabetadex, arimoclomol, acetyl-L-leucine, Adenovirus viral vector gene therapy (Preclinical animal studies only)
